# Values clarification in a decision aid about fertility preservation: does it add to information provision?

**DOI:** 10.1186/1472-6947-14-68

**Published:** 2014-08-09

**Authors:** Mirjam M Garvelink, Moniek M ter Kuile, Anne M Stiggelbout, Marieke de Vries

**Affiliations:** 1 Department of Gynecology, Leiden University Medical Center (LUMC), Mail zone VRSP, P/O Box 9600, 2300 RC Leiden, the Netherlands; 2 Department of Medical Decision Making, LUMC, Leiden, the Netherlands; 3 Tilburg Institute for Behavioral Economics Research (TIBER), Department of Social Psychology, Tilburg University, Tilburg, the Netherlands

**Keywords:** Values clarification method, Decision aid, Decisional conflict, Knowledge, Personality, Information seeking style, Experiment

## Abstract

**Background:**

We aimed to evaluate the effect of a decision aid (DA) with information only compared to a DA with values clarification exercise (VCE), and to study the role of personality and information seeking style in DA-use, decisional conflict (DC) and knowledge.

**Methods:**

Two scenario-based experiments were conducted with two different groups of healthy female participants. Dependent measures were: DC, knowledge, and DA-use (time spent, pages viewed, VCE used). Respondents were randomized between a DA with information only (VCE-) and a DA with information plus a VCE(VCE+) (*experiment 1*), or between information only (VCE-), information plus VCE without referral to VCE(VCE+), and information plus a VCE with specific referral to the VCE, requesting participants to use the VCE(VCE++) (*experiment 2*). In experiment 2 we additionally measured personality (neuroticism/conscientiousness) and information seeking style (monitoring/blunting).

**Results:**

*Experiment 1*. There were no differences in DC, knowledge or DA-use between VCE- (n=70) and VCE+ (n=70). Both DAs lead to a mean gain in knowledge from 39% at baseline to 73% after viewing the DA. Within VCE+, VCE-users (*n*=32, 46%) reported less DC compared to non-users. Since there was no difference in DC between VCE- and VCE+, this is likely an effect of VCE-use in a self-selected group, and not of the VCE per se. *Experiment 2*. There were no differences in DC or knowledge between VCE- (n=65), VCE+ (n=66), VCE++ (n=66). In all groups, knowledge increased on average from 42% at baseline to 72% after viewing the DA. Blunters viewed fewer DA-pages (R=0.38, *p*<.001). More neurotic women were less certain (R=0.18, *p*<.01) and felt less supported in decision making (R=0.15, *p*<.05); conscientious women felt more certain (R=-0.15, *p*<.05) and had more knowledge after viewing the DA (R=0.15, *p*<.05).

**Conclusions:**

Both DAs lead to increased knowledge in healthy populations making hypothetical decisions, and use of the VCE did not improve knowledge or DC. Personality characteristics were associated to some extent with DA-use, information seeking styles with aspects of DC. More research is needed to make clear recommendations regarding the need for tailoring of information provision to personality characteristics, and to assess the effect of VCE use in actual patients.

## Background

### Preference sensitive decision making and decision aids (DAs)

An increasing number of medical decisions are preference sensitive, indicating that the “best” decision or treatment option does not only depend on what is best from a medical point of view, but depends on patient preferences with regard to the treatment options as well, and should therefore take into account the values a patient attaches to the advantages and disadvantages of those option(s). In other words, with preference sensitive decisions, patients should be actively invited to participate in decision making [1-3].

In order to increase participation in decision making and improve decision making processes and outcomes for preference sensitive decisions, decision aids (DAs) are increasingly used. DAs are tools that provide at minimum some information about the (medical) problem, possible solutions, including an option to wait and see, information about risks and uncertainties, and a balanced overview of advantages and disadvantages of each option [4].

Despite availability of quality criteria for the development and evaluation of DAs [5], which are used by most DA developers, DAs differ with regard to the type of medium (e.g. brochures, booklets, DVD’s, CD-ROMs, websites), their content, and the offered decision making support [6-8]. Some DAs provide patients with information only, summaries, or patient narratives, with which patients can implicitly clarify what is important for them. Others combine information with explicit values clarification methods (VCM), in which patients are supported in active deliberation about what is important to them.

In general, DAs as a whole have been found to be effective in reducing DC, to increase knowledge on the subject, to lead to more realistic expectations, and to lead to a higher percentage of patients who are able to decide on a course of action [3]. However, the effect of specific aspects, such as VCMs (if effective at all) is less clear [3,7,9-12]. Two patient studies that have evaluated the effect of DAs with several types of VCM compared to DAs without VCM or information only, did find that VCMs in the form of an explicit values clarification exercise (VCE) lead to a higher percentage of patients who made an informed decision that was in agreement with their personal values [3], a higher congruence between values and treatment [3], and lead to feeling better prepared for decision making [13]. Prior studies with healthy participants have found mixed results [7,10]. When comparing explicit with implicit VCM [7,12], explicit VCM were more effective in healthy participants [7], but no improvements were found in patient populations [12]. Additionally, in theory, deliberation (with VCM) and analytical reasoning may not always be beneficial for decision making [11], since deliberation may overshadow important intuitive feelings that are more difficult to formulate but may be just as important in decision making [11].

### The decision

A good example of a preference sensitive decision with a difficult decision making process is the decision whether or not to undergo fertility preserving procedures (fertility preservation, FP) before the start of the cancer treatment when diagnosed with breast cancer. The last decades, chemotherapy for breast cancer has increased survival chances, but with an increased possibility of losing fertility as a consequence [14]. Since many young cancer patients have a future child wish, interest has risen in possibilities to preserve fertility before undergoing cancer treatment. At this moment one can try to spare fertility by cryopreserving embryos, oocytes, or ovarian tissue [15]. However, since chances to become infertile are never 100%, not undergoing any fertility sparing treatment (wait and see) is also an option [14,16]. All these FP options come with risks and success rates [15,16]. For some years, FP has been offered to young women with breast cancer (18–40 years old). Not only are there many aspects to consider in deciding about FP, but the decision also has to be made in the short time frame (often a few days to a week) between diagnosis and start of the chemotherapy treatment, with competing demands from other breast cancer-related decisions and emotions [17].

**Figure 1:**
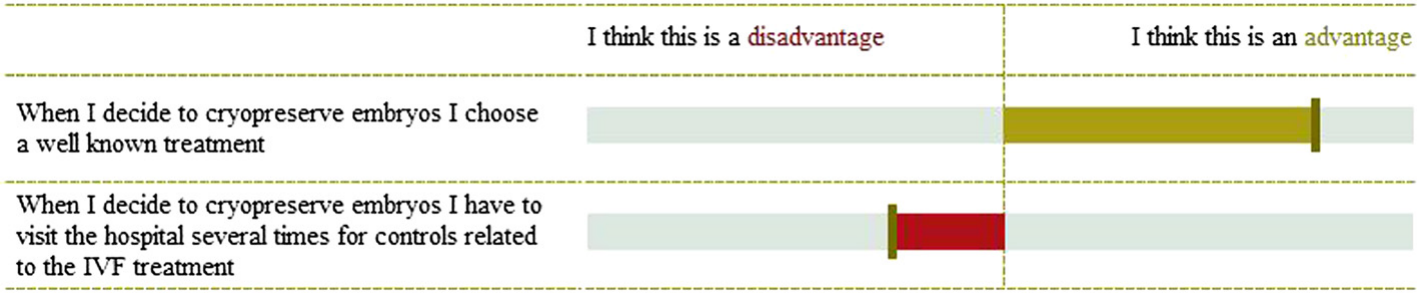
**Example of a statement in the value clarification exercise (cryopreservation of ovarian tissue).** For each statement in the value clarification exercise, patient rate whether it is an advantage (green; right side of the figure) or disadvantage (red; left side of the figure) and the extent to which the statement is considered important in decision making about FP (by lengthening or shortening the bar) [18].

**Figure 2:**
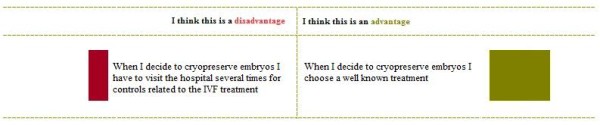
**Example of the summary of given ratings.** The *red* box in the column with *disadvantages* (left side of the figure) represents the extent to which the rated disadvantage is important in the decision whether or not to opt for a certain FP option (in this case cryopreservation of embryos), as indicated by the patient herself in the previous step (Figure 1). The *green* box in the column with *advantages* (right side of the figure) represents the extent to which the rated advantage is important in the decision whether or not to opt for a certain FP option (in this case cryopreservation of embryos), as indicated by the patient herself in the previous step (Figure 1) [18].

In order to assist decision making about FP, we have developed a DA for women with breast cancer who have to decide about FP treatments [18]. The DA has been designed for use by individual patients, as preparation for a consultation with a clinician in which the final decision is made. The DA consists of textual information (Table 1), and a fine-grained, explicit VCE. The few studies that have evaluated the impact of VCEs found indications for a beneficial effect of adding a VCE to a DA with regard to the percentage of patients who made an informed decision that was in agreement with their personal values [3,12]. We have chosen for an explicit VCE since explicit VCEs showed indications of being more effective than implicit VCEs with regard to satisfaction with preparation for decision making [13] and decisional conflict [7]. We have chosen a fine grained, additive VCE (comparing attributes of one treatment option at a time) [9], as we wanted patients to choose only between pursuing (or not) the options for which they are eligible. Each VCE consists of statements about the consequences of a FP option, for each of which patients are asked to indicate the extent to which they were considered a benefit or disadvantage (Figures 1 and 2). Patients indicated on two VAS scales a) whether the statement is considered to be an advantage or disadvantage to the FP option, and b) the importance of the statement [9] (Figure 1). Additionally, patients have the option to add arguments and rate these as well. After rating the importance of the separate statements, the DA generates a summary that provides an overview of patients’ answers in descending order from most important to least important (as indicated by the patient) (Figure 2). Moreover, patients can indicate the extent to which they are in favor of the treatment options, and make a decision based on their own values. Patients are not provided with a clear-cut advice about which treatment to choose. This overview was chosen, rather than a summary bar indicating how much someone favors one of the FP treatments [9], because we did not want to steer patients towards one of the treatments. In previous studies with this DA, acceptability, comprehensibility and user-friendliness were assessed in patients and clinicians and both the textual information and the VCE were considered relevant, coherent and understandable [18]. We hypothesized that the use of our DA with VCE in deciding about FP would decrease DC compared to information only [7,13].

**Table 1:** **Content of the web-based decision aid “Breast cancer and wish for children” [**[18]**]**

**Chapter #**	**Titles and subtitles**
1.	**Can I still achieve a pregnancy (after my treatment for breast cancer)?**
	• Chemotherapy
	• Hormonal therapy
	• Other treatments
2.	**What can I do now to be able to have children later?**
	• Wait and see
	• Cryopreservation of embryos
	• Cryopreservation of ovarian tissue
	• Cryopreservation of oocytes
3.	**What if I cannot achieve a pregnancy later?**
	• No children
	• Oocyte donation
	• Adoption
	• Foster parenting
4.	**Background information**
	• Fertility
	• Pregnancy and breast cancer
	• Genetics and breast cancer
5.	**Deciding about fertility preservation**
	• What is important to me?
	- Wait and see
	- Cryopreservation of embryos
	- Cryopreservation of ovarian tissue
	• Question prompt list

Emotions, coping styles and personal characteristics may influence decision processes and the extent to which informational sources are used [19-22]. Since patients may react with feelings of anxiety and depression to the news about a diagnosis with a life threatening disease such as breast cancer and the prospect of a fertility threatening cancer treatment [23-25], it may be important to acknowledge these emotions. Furthermore, emotions may affect values related to the decision, and risk perception [26]. Additionally, patients may have their own coping styles when it comes to getting informed about threatening medical situations, which is reflected in their preferred role in decision making and consequently their behavior with regard to seeking information. For example, patients with monitoring coping styles have been found to ask more questions in the consultation, and to prefer more detailed information [27]. Moreover, it has been suggested that patients with a more neurotic personality prefer less participation in decision making about treatment, while more conscientious patients prefer more participation and deliberation [28]. We therefore hypothesized that having a monitoring coping style or a more conscientious personality would be associated with more extensive use of the DA and VCE, less DC, and more knowledge after viewing the DA. Blunting coping styles and neurotic personalities were thought to be associated with less use of the DA and VCE, more DC and less knowledge after viewing the DA.

### The current research

In order to test the above mentioned hypotheses, two experiments were performed with healthy participants making hypothetical decisions about FP. In order to make participants more similar to patients, we induced with neutral, sad and anxious emotions in them. Whereas we are well aware of the limitations of including healthy participants instead of patients, we chose for healthy participants to be able to include enough participants to reach sufficient power. Additionally, we thought it would be unethical to test these specific hypotheses in a patient population, before they were tested in non-patients.

In experiment 1 we studied the effect of type of DA (information only versus information + VCE) on DA-use, DC, and knowledge. Additionally we assessed the effect of VCE-use on DC and knowledge. In experiment 2 we assessed associations between several personality characteristics and information seeking styles with the extent to which the DA was used and on DC and knowledge.

## Experiment 1

### Methods experiment 1

#### *Study design*

**Figure 3:**
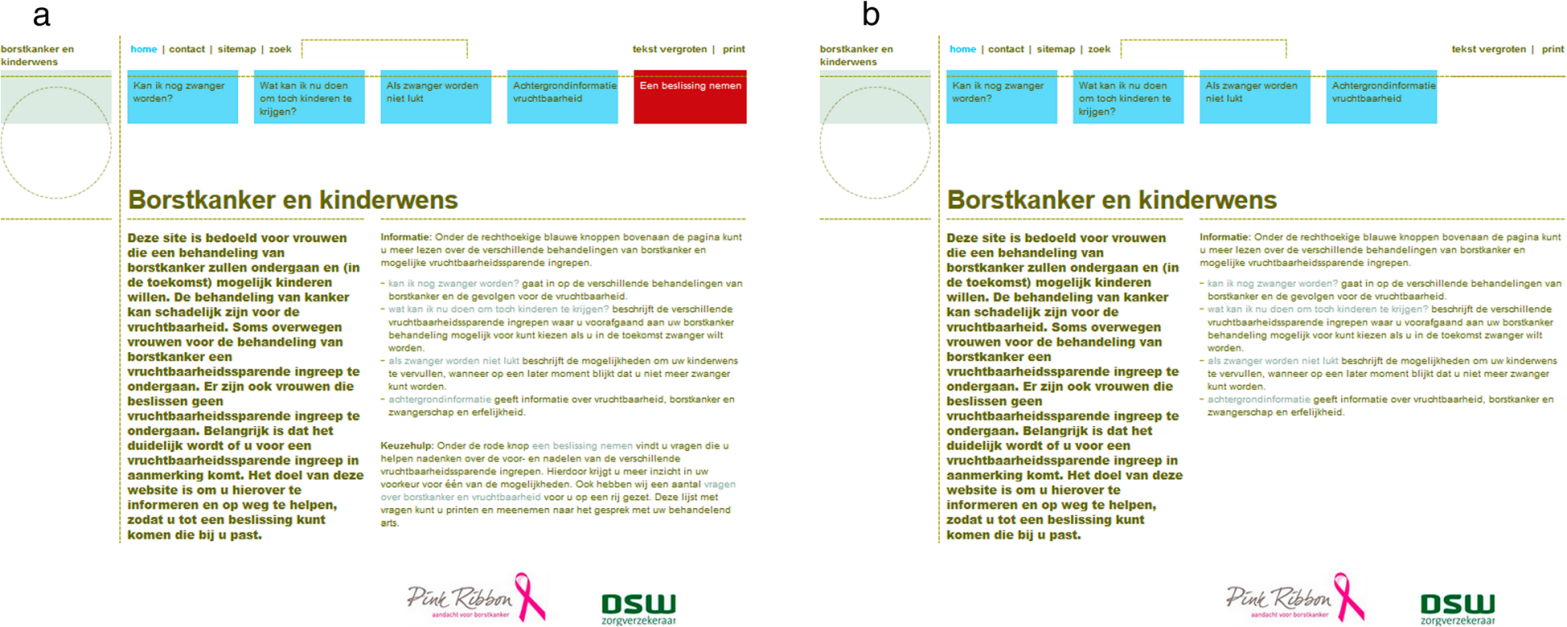
The two types of DAs: a) DA with information (blue buttons) and the VCE (red button); b) DA with information only (blue buttons).

The study was a 2 (type of DA: DA with information only or DA with information and a VCE) by 3 (emotion: neutral, anxious, or sad) between subjects factorial design, stratified by location (Leiden University – location 1, Tilburg University – location 2). For the randomization we used a block randomization scheme with variable blocks sizes containing all 6 possible combinations of emotions and type of decision aid randomization per block, developed by the department of medical statistics of the LUMC. The DA with information only consists of textual information (consisting of 20 separate webpages; for lay out see Figure 3b) and the DA with VCE additionally consists of a VCE for each FP option (consisting of six separate webpages) (for lay out see Figure 3a). All participants gave their informed consent before participating. The experiment has been performed in accordance with the Declaration of Helsinki. Experiment 1 was primarily conducted at location 2, where no formal ethical approval was required.

#### *Participants*

Participants (N = 140) were healthy women between 18–36 years old (*M* = 20.8, *SD* = 3.4), who had sufficient understanding of the Dutch language. Participants were invited by advertisements at universities, in libraries and on websites (including social media). Participants participated in exchange for either money (location 1; 8 euros) or course credits (location 2). Participants at location 1 had to actively approach the researcher and had to make an appointment to participate. Participants at location 2 could easily subscribe through an online system. All participants gave their informed consent before participating.

#### *Procedure*

##### 

**Measurements** The study was completely computerized, outcomes were measured with questionnaires and web statistics. All measures were measured immediately after viewing the DA, except for knowledge which was measured both before and after viewing the DA.

The primary outcome measure was DC. This was measured with a Dutch translation of the decisional conflict scale (DCS) (including the subscales values clarity, informed decision making, effective decision making, decision making support, decision making uncertainty) [29]. The total scale consists of 16 items measured on a 5 point Likert scale ranging from 0 (totally disagree) 4 (totally agree). A total DC score is obtained by adding up the scores on the items, dividing them by the number of items and rescoring them from 0–100. A higher score on the DCS, or one of its subscales, indicates more DC.

Other outcomes were knowledge about FP, measured with 10 statements about FP options with the answer categories “true”, “false”, or “I do not know”. Furthermore, we measured preferred FP option (5 categories: wait and see (not undergoing a fertility sparing treatment), cryopreservation of embryo’s, oocytes, ovarian tissue, do not know), socio-demographic characteristics (age, child wish, parity, experience with (breast) cancer in relatives and peers, relational status, cohabiting, education, ethnicity, religious affiliation), and web statistics such as total time spent on the DA and number of informational- and VCE-pages viewed.

##### 

**Emotion induction** Emotions were induced by a combination of a short film fragment and background music during the entire experiment, two methods that have previously been found to be successful for inducing moods [30].

Directly after emotions were induced, respondents read a neutral, sad or anxious hypothetical script (subtly adapted to the induced emotions with words related to how somebody feels) in which they were asked to imagine that they were at a consultation with their oncologist and just received the diagnosis of breast cancer, for which they would be treated with chemotherapy. Since chemotherapy might influence their fertility, they are offered the chance to preserve their fertility before undergoing chemotherapy. At the end of the script women were referred to a DA website to prepare them for making a decision. Respondents were then actually referred to the DA, using the following text: “by clicking on the link below you are referred to a decision aid about fertility preservation for breast cancer patients. You are asked to make a decision whether or not you want to preserve your fertility, and if so, how”. They were instructed to spend as much time, and view as many pages on the DA as they thought was necessary to make a decision, there was no minimum or maximum.

In order to test whether the emotion induction was successful, participants were asked before (pre induction - I), immediately after emotion induction and after reading the script (post induction - II), and after viewing the DA (post DA - III), to what extent they felt happy, anxious and sad at that moment on a 7-point Likert scale (i.e. “to what extent do you feel happy at this moment?”). This emotion manipulation check indicated that all participants felt more sad (Δ*M* = 2.1) and anxious (Δ*M* = 2.1) after induction, and less happy (Δ*M* = -2.0). No differences were observed between the three emotion induction conditions. Likely, the hypothetical script, which all participants had to read following the emotion induction and before measurement of emotions, and the decision itself, may have evoked feelings of sadness and anxiety in all participants. Since no differences on perceived emotions were found between emotion induction conditions, we controlled for emotion induction condition in all analyses but no further analyses were conducted with emotions.

#### *Statistics*

Analyses were conducted with SPSS 20.0. Differences between the DAs in continuous outcomes with only one measurement moment (e.g. DC) were tested with one-way ANOVAs with DA-type (VCE +/-) as between-subjects factor. Differences in knowledge scores at baseline and after viewing the DA were tested with a General Linear Model (GLM) for repeated measures, with DA-type (VCE +/-) as between-subjects factor.

Analyses were subdivided in primary (intention to treat) and secondary analyses (based on actual use of the DA and VCE). Since not all participants randomized to information plus VCE actually used the VCE, we conducted secondary analyses with a new grouping variable, consisting of three arms: information only (VCE-), information plus a VCE which was not used (VCE +-), and information plus a VCE which was used (VCE ++). This variable (three groups) replaced the fixed variable “DA-type” in the ANOVA and GLM for repeated measures as described above. Post hoc analyses were conducted to check for specific group differences. All the analyses were done, while controlling for the effect of emotion induction condition and location.

#### *Power calculation*

A sample size of 64 participants per treatment arm was considered sufficient to analyze main effects on DC with a power of 0.8 (Cohen’s *d* = 0.5; *β* = 0.2; *α* = 0.05). Within the two DA-conditions respondents were equally randomized among the three different emotion conditions.

### Results experiment 1

#### *Participants and socio-demographic characteristics*

One-hundred fifty-one women participated. We excluded 11 women because of incomplete data on main outcomes due to problems with internet or the questionnaire. The total population used for data analyses consisted of 140 participants, 39 in location 1, and 101 women in location 2.

At baseline there were no differences in socio-demographic characteristics between the locations (data not shown). Furthermore, randomized conditions (DA-types) were comparable on most socio-demographic characteristics. With regard to future desire for children we found that women in the information only condition somewhat less often had a child wish than women in the VCE + conditions (*χ*^*2*^ = 7.17, *p* < .05; Table 2).

**Table 2:** Socio-demographic characteristics, differences in decision-making, decisional conflict, knowledge and DA use between women who received a DA with information only or a DA with information and an explicit VCE (subdivided by whether they used the VCE or not), controlled for emotion induction condition

	**DA with information only (VCE-)**	**DA with information plus VCE (VCE+)**			**Primary analysis (A vs B)**		**Secondary analysis (A vs C vs D)**	
	**Total group VCE- (N = 70)**	**Total group VCE + (N = 70)**	***VCE used (VCE++) (n = 33)***	***VCE not used (VCE + -) (n = 37)***	**F-(condition) or χ2-value**	**Post hoc**	**F-(condition) or χ2-value**	**Post hoc**
	**A**	**B**	***C***	***D***				
Age, mean (SD)	20.7 (3.3)	20.9 (3.5)	*20.4 (3.5)*	*21.6 (3.5)*	NS		NS	
Child wish, yes, n (%)	56 (80)	64 (91)	*34 (91)*	*30 (91)*	6.9*	A < B	7.17*	A **<** D = C
Children, yes, n (%)	3 (4)	-	*-*	*-*	-		-	
Partner, yes, n (%)	34 (49)	42 (60)	*24 (65)*	*18 (55)*	NS		NS	
**Decisional conflict**								
Total DCS, M (SD)	40.9 (11.6)	43.6 (14.2)	*37.9 (15.7)*	*48.6 (10.6)*	NS		6.4**	A = C **<** D
Values clarity, M (SD)	27.7 (14.5)	32.0 (18.4)	*22.7 (16.4)*	*40.3 (16.1)*	NS		9.4**	A = C **<** D
Decisional support, M (SD)	44.7 (14.2)	45.9 (16.7)	*38.4 (18.2)*	*52.7 (11.9)*	NS		3.4*	A = C **<** D
Effective DM, M (SD)	32.8 (15.7)	33.6 (18.7)	*27.3 (19.9)*	*39.2 (15.9)*	NS		4.4*	D **>** C
Uncertainty, M (SD)	36.6 (17.8)	40.3 (16.8)	*40.9 (20.3)*	*39.8 (13.2)*	NS		0.97	NS
Informed DM, M (SD)	65.2 (22.6)	69.6 (22.9)	*64.1 (26.6)*	*74.5 (17.9)*	NS		3.2*	A = C **<** D
**Knowledge**								
Knowledge post DA, M (SD)	7.3 (1.9)	7.2 (1.7)	*7.4 (1.7)*	*6.9 (1.8)*	NS		NS	
**Time spent (minutes)**								
Total time spent, M (SD)	8.5 (7.4)	9.3 (8.4)	*14.3 (9.2)*	*4.9 (4.4)*	NS		9.2**	D < A < C
Time spent on information only, M (SD)	8.5 (7.4)	8.3 (7.3)	*11.8 (8.0)*	*4.9 (4.4)*	NS		3.9*	D < A < C
Pages viewed Δ	12.5 (2–38)	14.4 (3–36)	*21 (9–36)*	*8.5 (3–17)*	NS		27.61**	D < A < C
Made a decision, yes, n (%)	56 (80)	58 (82.8)			NS		NS	

* = p < 0.05 ** = p < 0.01, NS = not statistically significant; M = mean; SD = standard deviation; A = DA with information only (VCE-); B = DA with information and VCE (VCE+); *C = DA with information and VCE, VCE used (VCE++). D = DA with information and VCE, VCE not used (VCE + -)*. Δ some pages were viewed repeatedly by the respondents.

##### 

**Effect of type of DA on decision making, DA use, decisional conflict, knowledge (Primary analyses)** Of the total population, 114 women (81%) were able to make a decision whether or not to preserve fertility: 24 women (21%) wanted to wait and see, and 90 women (79%) chose to cryopreserve either embryos (*n* = 45), oocytes (*n* = 34) or ovarian tissue (*n* = 11).

There were no effects of DA-type (information with or without VCE) on time spent on the DA or number of pages viewed (Table 2). Mean number of pages viewed for the total group was 13.4 (*SD* = 7.7) and mean time spent on the DA was 8.9 minutes (*SD* = 7.9). The correlation between time spent on DA and pages viewed was high (*r* = .75, *p* < .001), therefore we chose to use only “time spent” in further analyses.

There were no significant differences in DC (including scores on all subscales) or knowledge between women who received the DA with information only (VCE-) or with information and a VCE (VCE+) (Table 2). In both conditions, the DA led to a significant increase in knowledge (*F*(1,127) = 264.96, *p* < .001). At baseline, mean knowledge score for the total group was 4.2, after viewing the DA it was 7.6; a relative increase of 81%. Moreover, after adjustment for baseline knowledge there was a significant positive relation between knowledge after viewing the DA and time spent on the DA (*r* = .33 *p* < .001).

Since there was a significant difference in desire for children between the groups, we have repeated all the analyses correcting for desire for children. Results of the additional analyses were similar to the above mentioned results.

##### 

**Effect of using the VCE on total DA use, decisional conflict (Secondary analyses)** Of the women in the VCE + condition (*n* = 70), only 33 women (47%) had viewed the VCE (VCE++, Table 2). These women spent on average 2.5 minutes (range 10 seconds – 8 minutes) on the VCE. There was a significant difference in time spent on the DA between women who did or did not use the VCE, but not with women who were not able to use the VCE (*F*(2,123) = 9.2, *p* < .001). Women who had used the VCE spent more time on the DA than women who had not.

Posthoc analyses indicated that women who used the VCE, and women who received information only (who were not able to use a VCE), reported significantly better (lower) scores on DC (*F*(2,122) = 6.4, *p* < .01), values clarity (*F*(2,122) = 9.4, *p* < .001), decisional support (*F*(2,122) = 3.4, *p* < .05), and informed decision making (*F*(2,122) = 3.2, *p* < .05) than women who were able to but did not use the VCE. Furthermore, women who had used the VCE reported better (lower) scores on effective decision making (*F*(2,122) = 4.4, *p* < .05) than those who did not use it (Table 2).

### Conclusion experiment 1

Experiment 1 showed no difference in knowledge or DC between women who received a DA with or without a VCE. Secondary analyses revealed less DC for women who *used* the VCE compared to those who chose not to use it. However, there was no difference in DC between the VCE-users and the women who received a DA with information only (without VCE).

## Experiment 2

Experiment 1 showed less DC for women using a VCE compared to women who chose not to use it, but no difference compared to women who received information only, so we were interested in finding explanations for this difference. Since there was no difference between the VCE-users and the women who received a DA with information only (without VCE) this might be an effect of VCE-use in a self-selected group (for example related to personality characteristics), and is not likely an effect of the VCE per se. Therefore, in experiment 2 personality characteristics were measured to investigate whether DA- and VCE-use and effectiveness of DA- and VCE-use were associated with certain personality characteristics.

In the first experiment, only a minority of respondents who received a DA with VCE, accessed the VCE. Since no emphasis was put on the availability of the VCE in their DA, it is possible that some did not see the VCE. Therefore, to increase the number of VCE-users in Experiment 2, we added a third condition to the experiment: information plus VCE, with explicitly referring to the VCE.

### Methods experiment 2

#### *Study design*

Participants (N = 199) were randomly assigned to a DA with information only (VCE-), a DA with information and a VCE without referring to the VCE (VCE+), and a DA with information and a VCE with explicitly referring to the VCE (VCE++), stratified by location (Leiden University – location 1, Tilburg University – location 2). For the randomization we used a block randomization scheme with variable blocks sizes containing all 3 possible combinations of type of decision aid and referral to the VCE per block, developed by the department of medical statistics of the LUMC. Respondents were referred to the DA with the following text: “By clicking on the link below you are referred to a decision aid about fertility preservation for breast cancer patients. You are asked to make a decision whether or not you want to preserve your fertility and how”. Respondents who were specifically referred to the VCE additionally received the following text: “We would like to point out that the decision aid consists of both textual information about fertility preservation as well as the chapter “deciding about fertility preservation” which is meant to help you order your thoughts about fertility preservation and make a decision. Please use this chapter in making your own decision about fertility preservation”. The experiment has been performed in accordance with the Declaration of Helsinki. The study was approved by a local Ethics Committee at Leiden University, from Tilburg University no additional formal ethical approval was required.

#### *Participants*

Participants were healthy women between 18–32 years old (*M* = 21.4, *SD* = 2.3), with sufficient understanding of the Dutch language. Participants were invited by advertisements at the same universities as in experiment 1. Participants participated in exchange for either course credits/hours or money (6 Euros) at both study locations. All participants gave their informed consent before participating.

##### 

**Procedure** The study consisted of two parts. Part I consisted of completing questions about personality and information seeking style. Part II consisted of reading a neutral hypothetical script (see Experiment 1) after which respondents viewed a version of the DA (according their randomization) and completed questionnaires related to their decision making (process). Both parts were presented as independent studies of different researchers.

##### 

**Measurements** Measures were as in experiment 1, with addition of the following scales:

*Information seeking styles* (Monitoring and Blunting) were measured with a short version of the Threatening Medical Situations Inventory (TMSI) of Miller [31], after the example of Ong et al. [27]. A monitoring information seeking style indicates cognitive confrontation; a person with this style tends to actively seek out and monitor information about the threatening event [31]. A blunting information seeking style indicates cognitive avoidance; a person with this style tends to seek cognitive distraction from the threatening event and psychologically blunts threat-relevant information [31]. Respondents were asked to read two hypothetical situations (1-vague suspicious headache complaints and 2-choosing for uncertain heart surgery) and complete three monitoring and three blunting items on a five point Likert scale ranging from 1–5 (not at all to strongly applicable to me) for each scenario. Total monitoring and blunting scores were calculated by adding up all relevant items.

*Personality traits* (neuroticism and conscientiousness) were measured with the neuroticism (8 items) and conscientiousness subscales (9 items) of the Dutch translation of the Big Five Inventory [32]. A high score on neuroticism indicates that women are emotionally instable; a high score on conscientiousness indicates that women are well-organized and task- and goal-directed [33]. Participants were asked to rate their agreement with statements about their perception of themselves in varying situations, on a five-point Likert scale ranging from 1 (strongly disagree) to 5 (strongly agree). Total scores were calculated by adding up all relevant items, divided by the total number of items per scale.

#### *Statistics*

Differences in knowledge scores at baseline and after viewing the DA were tested with a General Linear Model (GLM) for repeated measures. Differences in other continuous outcomes were tested with ANOVAs. Analyses were subdivided in primary (intention to treat) and secondary analyses (based on actual use of the DA and VCE). Associations between personality characteristics and DA-use were studied with Pearson’s product moment correlations (PPMC) and GLMs.

All the analyses were done, while controlling for the effect of location.

#### *Power calculation*

Presuming a medium effect size (*f* = 0.25), we needed a total of 179 participants in three groups to reach a power of 0.8 (*α* = 0.05, *β* = 0.2, with 1 covariate).

### Results experiment 2

#### *Participants and socio-demographic characteristics*

One hundred ninety-nine eligible women participated. Due to missing data on some questions, the total population used for data analyses consisted of 197 participants, 91 women in location 1, and 106 women in location 2.

At baseline, there were no significant differences with regard to socio-demographic characteristics between conditions. Mean age of the respondents was 21.4 years old (range 18–32), 179 women (90%) had a future desire for children, and nobody had children.

#### *Effect of condition on decision making, DA use, decisional conflict, knowledge*

##### 

**Primary analyses**One hundred fifty-two women (77%) were able to make a decision whether or not to preserve fertility, of which 31 (20%) women wanted to wait and see, and 121 (80%) women chose to cryopreserve either embryos (*n* = 67), oocytes (*n* = 47) or ovarian tissue (*n* = 7).

There were no differences between the 3 conditions in total time spent on the DA and the extent to which the informational pages were used (Table 3). However, we did find differences in the extent to which the VCE was used; women who were referred to the VCE significantly more often used the VCE (*F*(2,127) = 3.2 *p* < .05), viewed more VCE pages (*F*(2,127) = 8.8, *p* < .001), and spent more time on the VCE (*F*(2,127) = 5.1, *p* < .01) than women in the VCE + condition who were not referred. No significant differences were found between randomization conditions with regard to DC (or subscales of the DCS) (Table 3). Use of the DA lead to a relative increase in knowledge of 71% (*M* = 4.2 to *M* = 7.2) in the total population (*F*(1,191) = 24.5 *p* < .001). No differences in knowledge were found between the randomization conditions. Moreover, after adjustment for baseline knowledge score there were significant positive relations between knowledge after viewing the DA, time spent on the DA (*r* = .33 *p* < .001), time spent on the informational pages (*r* = .31, *p* < .001) and time spent on the VCE (*r* = .18, *p* < .05).

**Table 3:** Differences in decision-making, decisional conflict, knowledge and DA use between women who received information only or information plus VCE (subdivided by referral to the VCE, and use of the VCE) (N = 197)

	**Information only VCE- (N = 65)**	**Information plus VCE (VCE+) (N = 132)**										
	**A**	**No referral to VCE (n = 66)**			**With referral to VCE (n = 66)**					**Primary analysis (A vs B vs E)**	**Secondary analysis (H vs I)**	
		**Total (no referral) (N = 66)**	***VCE not used (VCE + -) (n = 31)***	***VCE used (VCE++) (n = 35)***	**Total (with referral) N = 66**	***VCE not used (VCE + -) (n = 17)***	***VCE used (VCE++) (n = 49)***	**Total VCE not used *(C + F) *** **(n = 48)**	**Total VCE used *(D + G) *** **(n = 84)**	**F- value**	**F- value**	**Post hoc analysis**
		**B**	***C***	***D***	**E**	***F***	***G***	**H**	**I**			
Time spent (min)	7.7 (5.6)	8.9 (6.6)	*6.4 (6.5)*	*11.2 (6)*	9.4 (6.9)	*4.8 (5.1)*	*11.1 (6.8)*	5.8 (6.0)	11.1 (6.4)	NS	15.6**	H < I
Time on informational pages	7.7 (5.6)	7.8 (5.93)	*6.4 (6.5)*	*8.9 (5.2)*	7.1 (5.6)	*4.8 (5.1)*	*7.9 (5.6)*	5.8 (6.0)	8.3 (5.4)	NS	4.3*	H < I
Pages viewed (incl vce pages)	13.3 (8.7)	16.1 (9.7)	*11.1 (4.8)*	*20.4 (10.9)*	17.4 (11.4)	*7.7 (3.6)*	*20.7 (11.5)*	9.9 (4.6)	20.5 (11.2)	NS	20.9**	H < I
Informational pages	13.3(8.7)	13.2 (6.1)	*11.1 (4.8)*	*15.1 (6.6)*	11.9 (6.9)	*7.7 (3.6)*	*13.3 (7.3)*	9.9 (4.6)	14.1 (7.0)	NS	7.1**	H < I
**Knowledge**												
After viewing the DA	7.5 (1.6)	7.1 (1.9)	*7.3 (1.9)*	*6.9 (2.0)*	7.2 (1.8)	*6.5 (2.4)*	*7.5 (1.5)*	7.0 (2.1)	7.2 (1.8)	NS	NS	
**Decisional conflict**												
Total DCS M (SD)	44.1 (12.3)	43.6 (11.4)	*41.8 (10.1)*	*45.2 (12.3)*	41.6 (9.5)	*44.5 (7.6)*	*40.6 (9.9)*	42.8 (9.3)	42.5 (11.2)	NS	NS	
Values clarity M (SD)	32.9 (14.7)	34.2 (15.2)	*33.6 (13.3)*	*34.7 (16.8)*	30.6 (13.7)	*37.7 (12.2)*	*28.1 (13.5)*	35.1 (12.9)	30.9 (15.2)	NS	NS	
Decisional support M (SD)	45.2 (14.4)	45.3 (14.5)	*43.3 (12.4)*	*47.1 (16.0)*	43.3 (12.4)	*48.5 (12.6)*	*41.5 (11.9)*	45.1 (12.6)	43.9 (13.5)	NS	NS	
Effective DM M (SD)	37.3 (16.7)	34.2 (14.3)	*31.6 (12.9)*	*36.6 (15.2)*	32.3 (13.3)	*32.7 (16.7)*	*32.1 (12.1)*	32.0 (14.2)	34.0 (13.6)	NS	NS	
Uncertainty M (SD)	41.5 (16.7)	40.9 (16.7)	*41.1 (17.2)*	*40.7 (16.5)*	39.6 (14.6)	*37.3 (13.8)*	*40.5 (14.9)*	39.7 (16.1)	40.6 (15.5)	NS	NS	
Informed DM M (SD)	66.0 (21.4)	66.5 (18.2)	*62.9 (20.4)*	*69.7 (15.6)*	65.4 (19.9)	*70.6 (15.9)*	*63.6 (21.0)*	65.6 (19.1)	66.2 (19.1)	NS	NS	
**Personality traits, high M (SD)**												
Neuroticism	3.1 (0.53)	3.0 (0.61)	*2.9 (0.57)*	*3.1 (0.64)*	3.1 (0.54)	*3.1 (0.62)*	*3.1 (0.52)*	2.9 (0.58)	3.1 (0.57)	NS	NS	
Conscientiousness	3.5 (0.54)	3.6 (0.64)	*3.6 (0.59)*	*3.5 (0.69)*	3.5 (0.63)	*3.4 (0.82)*	*3.5 (0.56)*	3.5 (0.68)	3.5 (0.61)	NS	NS	
Monitoring	19.7 (4.59)	19.9 (4.31)	*20.1 (4.36)*	*19.7 (4.30)*	19.7 (4.1)	*19.3 (4.36)*	*19.9 (4.08)*	19.8 (4.34)	19.8 (4.15)	NS	NS	
Blunting	18.6 (2.9)	17.8 (3.11)	*18.1 (2.58)*	*17.5 (3.53)*	18.1 (3.2)	*18.1 (3.49)*	*18.1 (3.17)*	18.0 (2.88)	17.9 (3.31)	NS	NS	

*p < 0.05; **p < 0.001, NS = not significant; min = minutes; M = mean; SD = standard deviation; DA = decision aid; VCE = values clarification exercise. **A** = DA with information only (VCE-); **B** = DA with information and VCE, without referral to VCE; *C = DA with information and VCE, without referral to VCE, VCE not used; D = DA with information and VCE, without referral to VCE, VCE used*; **E** = DA with information and VCE, without referral to VCE; *F = DA with information and VCE, with referral to VCE, VCE not used; G = DA with information and VCE, with referral to VCE, VCE used.*

##### Secondary analyses

Of the women in the VCE + conditions (with and without referral, *n* = 132), 84 viewed the VCE (63%). Women who made use of the VCE spent more time on the total DA (*F*(2,128) = 15.6 *p* < .001), and on the informational pages of the DA (*F*(2,128) = 4.3, *p* < .01) and viewed more informational pages (*F*(2,128) = 7.1, *p* < .001) than those who did not, indicating that they used the whole DA more thoroughly. Within VCE + (with and without referral), there were no significant differences in DCS or any of the subscales between women who did (VCE++) or did not use the VCE (VCE + -), indicating that VCE-use was not related to differences in DC between the conditions (Table 3). No differences in knowledge were found between women who did or did not use the VCE.

#### *Effect of personality characteristics and information seeking style on DA use, decision making, decisional conflict and knowledge*

Personality characteristics and information seeking styles were equally distributed (Table 3).

Blunting (with regard to information seeking) was associated with viewing less informational pages (*r* = -.38, *p* < .001) and less total pages (*r* = -.29, *p* < .001). None of the personality traits were significantly associated to the extent to which the DAs were used (time spent, pages viewed). With regard to DC, being more neurotic was associated with more decision making uncertainty (*r* = .18 *p* < .01), and decision making support (*r* = .15, *p* < .05) and being more conscientious was associated with less decision making uncertainty (*r* = -.15, *p* < .05). None of the information seeking styles were associated with aspects of DC.

Knowledge after viewing the DA was associated with a more conscientious personality (*r* = .15, *p* < .05) and a more monitoring information seeking style (*r* = .15, *p* < .05) (corrected for baseline knowledge).

## Discussion

In the above mentioned experiments we assessed the effectiveness of a DA with information only or with additional VCE with regard to knowledge and DC, and the effect of personality characteristics on DA use and effectiveness. Additionally, in secondary analyses we assessed differences in effect between women who did or did not use the VCE. Experiment 1 showed no difference in knowledge or DC between DAs with or without a VCE. Secondary analyses revealed less DC for women who used the VCE compared to those who did not use the VCE, but it was unlikely that the VCE had caused this difference, since there was no difference in DC between women who received information plus VCE and used the VCE and women who received information only. In experiment 2 personality characteristics were measured to investigate whether DA- and VCE-use and effectiveness were affected by personality characteristics. Experiment 2 confirmed that there was no association between VCE-use and DC or knowledge, and showed that information seeking style affected DA use (number of pages viewed), but not VCE-use. Personality traits were to some extent associated with aspects of DC. In both experiments there was a large knowledge increase of both DAs, indicating that the information in the DA is beneficial with regard to knowledge, especially for women who use the DA more thoroughly, highly conscientious women and women with more monitoring information seeking styles.

Since quality criteria for DAs anticipate on the addition of a VCM to DAs [32,34], but the results between studies on the effectiveness of VCM vary from beneficial to no (significant) effects [3,7,11-13], we thought it was important to study the effect of our DA plus VCE before implementing it in patient care. However, it seems that not all patients or participants tend to use a VCE when available. In both our experiments there were women who had used the information on the DA, but not the VCE. Although active referral to the VCE increased use of the VCE, independent of personality or information seeking style, still 17 women (15%) who were referred to the VCE did not use it (*experiment 2*). In the condition without referral about half of the women used the VCE in both experiments. A study with patients who were actually facing the decision to undergo FP found even lower percentages of patients (23%) that used their VCE [35,36]. Although VCE-use does not have to take much extra time (in our experiments: ±5 minutes), it is an extra effort in the already short time patients have to get informed and make a decision, so it should be considered whether active referral is appropriate. The hereby conducted experiments did not show a direct beneficial effect of VCE-use with regard to knowledge or DC. Therefore, we found no obvious reason to recommend *increasing* VCE-use by actively referring patients to it. Since other VCM were not always beneficial either, quality criteria should perhaps be more cautious regarding VCM recommendation as well [37].

We did find a beneficial effect of both DAs (with or without VCE) on knowledge, since use of the DA lead to a relative knowledge increase of 71-81% compared to baseline (*experiment 2 and 1* respectively), and time spent on the DA was related to knowledge increase after using the DA. It is likely that the increase in knowledge is mostly related to the informational pages.

None of the personality characteristics or information seeking styles were associated with VCE-use; information seeking styles were only associated with DA-use in general, and personality was only associated with DC. However, effect sizes were small (<.3). Consistent with the literature, women with more blunting coping styles viewed less pages on the DA website [27,38]. More neurotic women reported to be more uncertain about the decision. However, Case et al. [39] mention that information seeking style does not only depend on personality, but also on the threat and controllability that is experienced, and on the desired effect of the information [39]. I.e., information can be used to do something about a potential threat, or to be reassured that there is no threat [39]. Additionally, anticipated emotions that are imagined with potential outcomes of decision making may affect the decision [26]. It is possible that our healthy participants did not really experience the threat, or did not have a desired emotion (which should follow from decision making), which may have affected their information seeking style and their decision making process. Also, it is likely that actual patients are sadder than healthy participants, and therefore elaborate more on information [40,41]. However, in the current experiments we were not able to study this properly. It is possible that participants in experiment 1 were more similar to patients because of the sad emotions that were induced with them. Moreover, all participants in experiment 1 felt more sad and anxious after the induction with happy, sad or anxious emotions. The most plausible explanation therefore is that besides the three different mood induction techniques that were used in the study (a movie, music and suggestions in the script) all participants had to read a relatively sad hypothetical script and make a difficult (hypothetical) decision, which may have overruled the effect of the other mood induction techniques. Unfortunately, this precluded us from analyzing the DA effectiveness in different emotional states.

In these experiments, levels of DC were relatively high (worse) compared to other studies with patients [12,42-44] and healthy participants [10], but comparable to studies with healthy students as participants [7,45]. Possibly, in contrast to what we would have expected, not actually facing the decision made decision making harder. Moreover, most studies which assessed DC in patients studied primary treatment decisions, which are different decisions than the decision to undergo FP or not, which is an “extra” decision that has to be made in an emotionally challenging period between diagnosis and start of the oncologic treatment [46,47]. For patients it is often a decision between their chances for survival, and the extent of their desire for children taken into account their possibilities for FP (related to personal characteristics such as their age, or whether they have a partner) [48]; factors that often exclude some FP options and therefore might facilitate decision making. Likely, our healthy participants who were not actually facing the decision of FP did not take these factors into account which may have increased their DC scores. Additionally, students are highly educated and may therefore approach the decision more analytically compared to patients from the general population which may increase DC scores. Interestingly, other studies with actual patients [3,13] more often find beneficial effects of VCEs than studies with healthy participants [7,10]. This may also be related to discrepancies between the way DAs are designed and how they are used in healthy participants. It should be noted that the DA as used in the experiments was originally designed for patients, who use the DA in preparation for a consultation with a physician in which a final decision is made about FP. This consultation is often within a few days after diagnosis (and DA use). In the experiments, respondents had to decide directly after viewing the DA, without support from a physician. Hence, both the limited amount of available time and the lack of interaction about the decision may have influenced decision making for our participants. It is likely that in the experiments decisions were made consciously since they were made directly after viewing the DA. Actual patients may make more intuitive decisions, since they are distracted in the time between using the DA and visiting the physician to decide. Sometimes, decision making may improve when the decision is made after distraction, due to the so-called unconscious thought effect [11,49].

These results have to be interpreted with caution due to some limitations. The DA used in this study was originally designed for patients, who make the decision in consultation with a physician, not directly after viewing the DA, so results of a healthy population making the decision by themselves, directly after viewing the DA may not be completely generalizable to patients that are actually facing this decision. Moreover, effects were measured immediately after decision making, but it is possible that a DA has more effect on DC and preparation for decision making sometime after the decision is made [13]. Despite randomization, there was a significant difference in desire for future children between women who received a DA with VCE and those who received information only in experiment 1. Although all respondents had to imagine that they had a “hypothetical desire for children” for the future as part of the hypothetical script, their actual desire for children could have influenced decision making about fertility preservation. Therefore, all analyses were repeated while controlling for whether or not women had a desire for children. As the results of these analyses were very comparable to the results reported here, we may conclude that the results of experiment one are not critically dependent on baseline levels of desired children. In experiment 1, fewer women than expected used the VCE, which reduced our power. Therefore we added a third condition to the second experiment, in which women were actively referred to the VCE.

## Conclusions

The above mentioned experiments indicate that our DA about FP for breast cancer patients seems beneficial with regard to knowledge increase, but that the VCE does not seem to improve knowledge or DC. However, nor did use of the VCE seem to cause any harm, other than the time involved in completing it (which was acceptable). Additionally, it is important to understand that personality characteristics and information seeking style may be important factors in determining the extent to which DAs are used and helpful for women. It is of utmost importance that these findings are assessed in patients as well, since results may be different when actually facing the decision to preserve fertility.

## Abbreviations

DA: Decision aid; VCE: Values clarification exercise; VCM: Values clarification method; FP: Fertility preservation.

## Competing interests

The authors declare that they have no competing interests.

## Authors ’contributions

MG Conception and design, data collection, analysis and interpretation, drafting the manuscript, critically revising, final approval. MtK Conception and design, analysis and interpretation, drafting the manuscript, critically revising, final approval. AS Conception and design, analysis and interpretation, drafting the manuscript, critically revising, final approval. MdV Conception and design, drafting the manuscript, critically revising, final approval.

## Pre-publication history

The pre-publication history for this paper can be accessed here:

http://www.biomedcentral.com/1472-6947/14/68/prepub
